# Quality of Life of Cancer Patients during Chemotherapy in Indonesia: A Comparison of EORTC QLQ-C30 and EQ-5D-5L, Based on Patients' Characteristics

**DOI:** 10.1155/2023/9357299

**Published:** 2023-03-03

**Authors:** Dyah A. Perwitasari, Fredrick D. Purba, Susan F. Candradewi, Haafizah Dania, Lalu Muhammad Irham, Imaniar Noor Faridah, Bayu P. Septiantoro

**Affiliations:** ^1^Faculty of Pharmacy, Universitas Ahmad Dahlan, Yogyakarta 55164, Indonesia; ^2^Faculty of Psychology, Universitas Padjadjaran, Jatinangor 45363, Indonesia; ^3^Department of Pharmacy, Kariadi Hospital, Semarang 50244, Indonesia

## Abstract

One of the important outcomes to define the success of cancer treatment is the health-related quality of life (HRQoL) that can be measured using generic and/or specific instruments. Our study aims to define the cancer patients' HRQoL in some hospitals in Indonesia as measured by the European Organization for Research and Treatment for Cancer (EORTC QLQ-C30) and the EQ-5D-5L, to define the differences of cancer patients' HRQoL referring to patients' characteristics, and to explore determinants of cancer patients' HRQoL. We recruited 451 cancer patients using a cross-sectional design in two referral hospitals in Central Java, Indonesia, using the purposive sampling technique. All subjects, recruited from July 2020 to October 2021, met the inclusion criteria, namely, adult patients diagnosed with cancers in all stages who willingly participated in the study. The Indonesian value set was used to obtain the EQ-5D-5L index score. We further analyzed the data based on cancer stages and compared two questionnaires using independent t test. We highlighted that most of the cancer patients are female (69.4%), young (86%), and at advanced stages of cancer (54.1%). The physical and role functions and global health status of the cancer patients are poor, and the most severe symptom is fatigue. Moreover, most of them experience severe pain and perform daily activities with difficulties. Some patients' characteristics show significant influences on the HRQoL domains in both questionnaires (*p* < 0.05). Interestingly, both of the questionnaires have shown significant correlations between similar domains and revealed the poor HRQoL of advanced cancer patients (*p* < 0.05). Our study finds that cancer patients still have poor HRQoL in some domains. We suggest to the health providers that they apply education and psychological intervention to increase their HRQoL.

## 1. Introduction

In 2020, the cancer burden in Indonesia included the total cancer cases, total cancer deaths, cost per year, and projected lives saved per year by 0.13%, 0.08%, 1.50 USD per capita, and 15,000, respectively. The highest numbers of cancer incidence and mortality in 2018 in Indonesia were 16.7% and 11.0%, consecutively [1]. Treatment for cancer patients has impacted various aspects of their lives, including quality of life [2]. They may experience more than one treatment, such as surgery, radiotherapy, hormonal treatment, and chemotherapy. Radiotherapy and chemotherapy may cause adverse events that in turn might deteriorate their quality of life [3].

Various factors such as cancer stages, cognitive functions, pain, financial aspects, and concerns about the future may influence the cancer patients' HRQoL [4]. Another study has also found that physical functions, emotional functions, pains, and symptoms experienced in cervical cancer could significantly decrease the patients' quality of life [5]. Additionally, some patients' characteristics, such as widowhood status and body weight, are associated with their HRQol [6].

Most investigations have found that cancer patients' HRQoL is deteriorating during cancer treatments. This condition highlights the need for evidence-based education and psychological interventions to improve their condition during the treatment [7–11]. Valid and reliable questionnaires about cancer patients' HRQoL are available and have been adapted into various languages worldwide, including Indonesia. The two questionnaires widely used to investigate cancer patients' HRQol are the European Organization for Research and Treatment for Cancer (EORTC QLQ-C30) [8] and EQ-5D-5L [12]. HRQoL in this research is defined as an individual's or group's perceived physical and mental health over time measured by the European Organization for Research and Treatment for Cancer (EORTC QLQ-C30) and EQ-5D-5L [8]. The former deals with the cancer-specific quality-of-life questionnaires, and the latter deals with the generic ones.

The aim of this study is to compare the EORTC QLQ-C30 and EQ-5D-5L scores in cancer patients based on the patients' characteristics. We further measured all the patients using two established questionnaires, namely, EORTC QLQ-C30 and EQ-5D-5L, to define the differences in cancer patients' HRQol referring to some of the patients' characteristics. Finally, we explore the determinants of cancer patients' quality of life.

## 2. Materials and Methods

### 2.1. Subjects

We employed a cross-sectional design to examine 451 subjects treated in two hospitals: Dr. Kariadi Hospital, Semarang, and Prof. Dr. Margono Soekardjo Hospital, Purwokerto, from 2020 to 2021. The inclusion criteria were all adult (above 17 years old) patients diagnosed with cancer in all stages who willingly participated in this study. We use purposive sampling methods in this research. The cancer patients were excluded from the research subject if they were unconscious and had more than one complication.

### 2.2. Instruments

**Figure 1:**
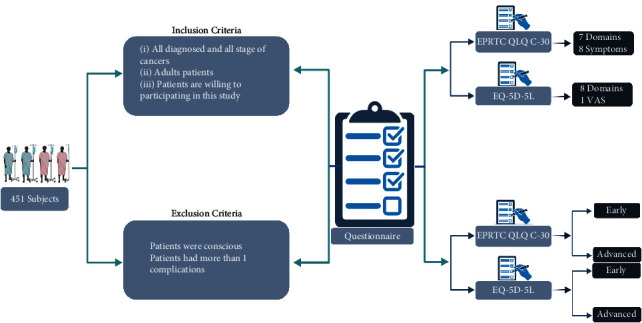
Workflow of the study.

The HRQol was measured using the EORTC QLQ-C30 and EQ-5D-5L. The EORTC QLQ-C30 is available in Bahasa Indonesia [8] and covers the following domains: physical, role-limitation, emotional, cognitive, social, and global health status. The EORTC QLQ-C30 measures the financial difficulty and symptoms caused by the cancer disease and/or treatments, such as nausea, vomiting, diarrhea, constipation, dyspnea, appetite loss, and insomnia. The EQ-5D-5L is a generic instrument to measure the HRQol and consists of five domains: mobility, self-care, daily activities, pain, and distress. Each domain has five levels of severity responses: no problems, slight problems, moderate problems, severe problems, and unable/extreme problems. Furthermore, this instrument identifies a visual analog scale (VAS) [13]. This instrument is also available in Bahasa Indonesia and has met the validity and reliability criteria [12]. The detailed workflow of the study is depicted in Figure 1.

The data of characteristics were collected from the cancer patients' medical records, and these characteristics include age, body weight, body height, cancer types, and cancer stages. The clinical data about the functional status and VAS score were collected from the questionnaire of EORTC QLQ-C30 and EQ-5D-5L directly from the patients' presence. All patients proceeded with the informed consent procedure. Our study was approved by the Ethical Committee of Prof. Dr. Margono Soekarjo Hospital No. 420/05887/2021 and the Ethic committee of Dr. Kariadi Hospital No. 401/EC/KEPK-RSDK/2019.

The univariate analysis was performed to describe the patients' characteristics and HRQoL. The independent *t*-test was calculated to define the differences in some domains by referring to the determinant categories. The correlation analysis was conducted to define factors influencing patients' HRQoL. Finally, all the analyses were conducted using SPSS version 21.

The principals for scoring EORTC QLQ-C30 were used based on the manual guidelines of EORTC QLQ-C30 [14]:

(1) Estimate the average of the items that contribute to the scale based on the manual scoring guidelines of EORTC QLQ-C30.
(2) Use linear transformation to standardize the raw score: not at all: score 1; a little: score 2; quite a bit: score 3; and very much: score 4. The transformation score is presented in Table 1.
  (1) $$\begin{array}{c} RawScore\left(RS\right):\frac{\left(I_{1}+I_{2}+\ldots +I_{n}\right)}{n}\text{.} \end{array}$$

The index value of EQ-5D-5L used in this research is based on the Indonesian EQ-5D-5L value set by Purba et al. in 2017. In this Indonesian value set, the highest index value is 1.000 for full health (“no problems in all five dimensions”) and the lowest is −0.865 for the worst health state (“unable/extreme problems in all five dimensions) [12].

## 3. Results

We recruited 451 cancer patients consisting of 69.4% of females, 86% of young patients, 54.1% of patients in an advanced cancer stage, and 32.4% of elementary school graduates. Most of the patients subsequently have no particular jobs (61.9%), earn less than 2.500.000 IDR (56.3%), and are married (87.6%).  Table 2 presents detailed information about the patients' characteristics.

Table 3 depicts various cancer types of 451 research participants. This study has highlighted the finding that the most cancer types are breast cancer and colorectal cancer (35.7%), followed by nasopharyngeal cancer (10%), lymphoma (3.5%), and cervical cancer (3.3%).

Table 4 presents the cancer patients' quality of life measured by the EORTC QLQ-C30. The higher the domain's score is, the better the patient's condition is. The domain's worst score is the role function (mean: 65.56 and SD: 33.79), and the domain's best score is the cognitive function (mean: 89.43 and SD: 15.98). The worst symptoms experienced by the cancer patients are fatigue (mean: 39.42 and SD: 2.52), followed by pain (mean: 36.11 and SD: 3.81) and insomnia (mean: 34.96 and SD: 3.71). The global health score is 68.05 (SD: 20.9).

Table 5 presents the proportion of responses in every domain of EQ-5D-5L. The majority of the patients have reported no problems with mobility, self-care, or anxiety/depression domains. However, 57.1% of the patients report that they have problems, ranging from slight to extreme problems, performing usual activities. Meanwhile, one-third of the patients (68.6%) report that they have experienced some levels of pain/discomfort.

**Figure 2:**
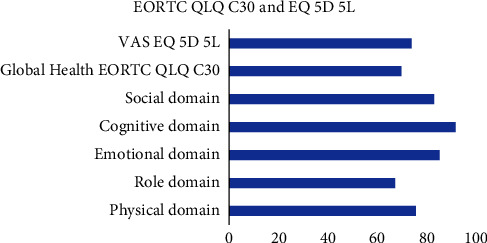
The global health score and other domains of the EORTC QLQ-C30 and VAS of EQ-5D-5L.

The utility index of the EQ-5D-5L is 0.68, and the EQ-VAS score is 72.09 or higher than that in the EORTC QLQ-C30 (68.05). This study has revealed a significant difference between the global health of QLQ-C30 and the VAS of EQ-5D-5L (*p* < 0.05). The comparison of the global health score and other domains of the EORTC QLQ-C30 and VAS of EQ-5D-5L is presented in Figure 2.

**Figure 3:**
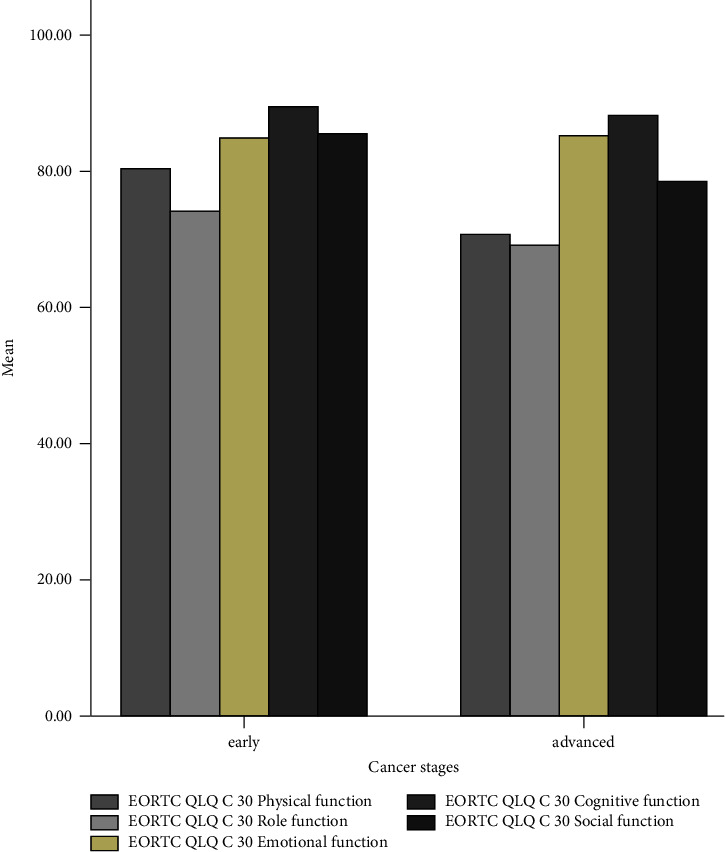
HRQoL domains based on cancer stages.

**Figure 4:**
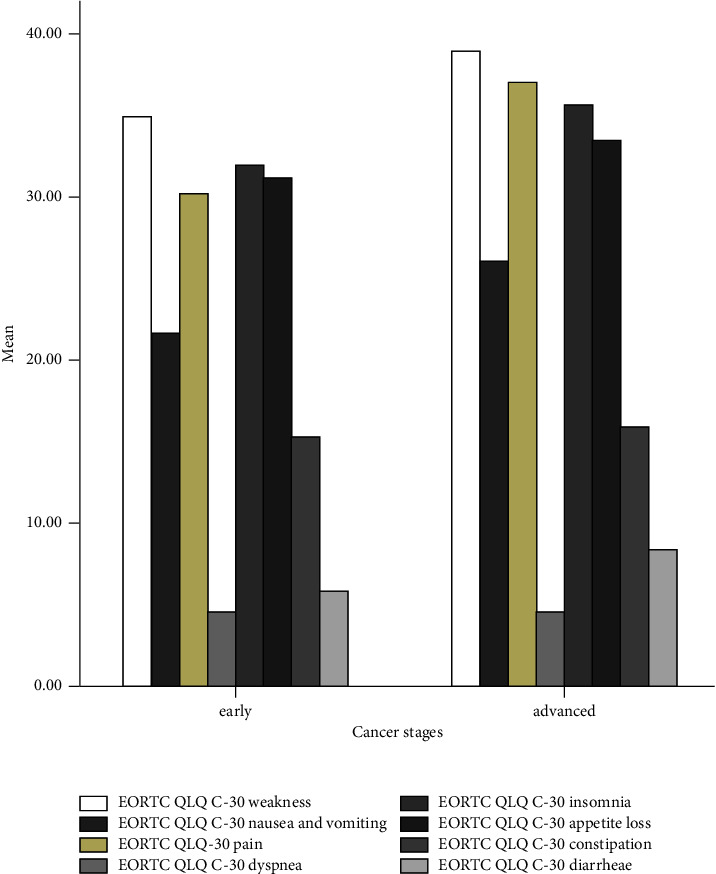
Symptoms during chemotherapy based on cancer stages.

Figure 3 depicts that all quality-of-life domains decrease in the advanced stages of cancer. This description can also be found in Figure 4 which shows that the patients in the advanced stage of cancer have experienced more severe symptoms.

**Figure 5:**
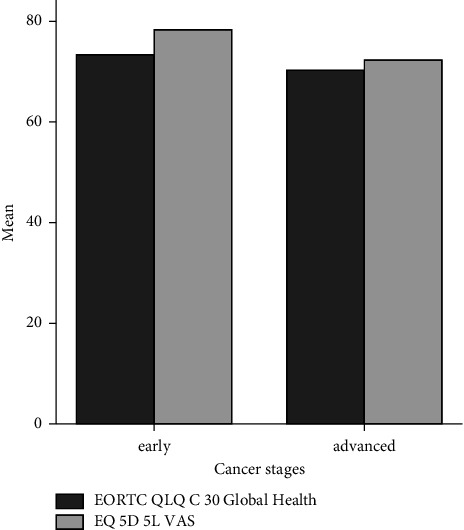
Scores of global health and VAS based on the cancer stages.

Figure 5 presents the scores of global health and VAS based on the cancer stages. The scores in both domains decrease in the advanced stages.

**Figure 6:**
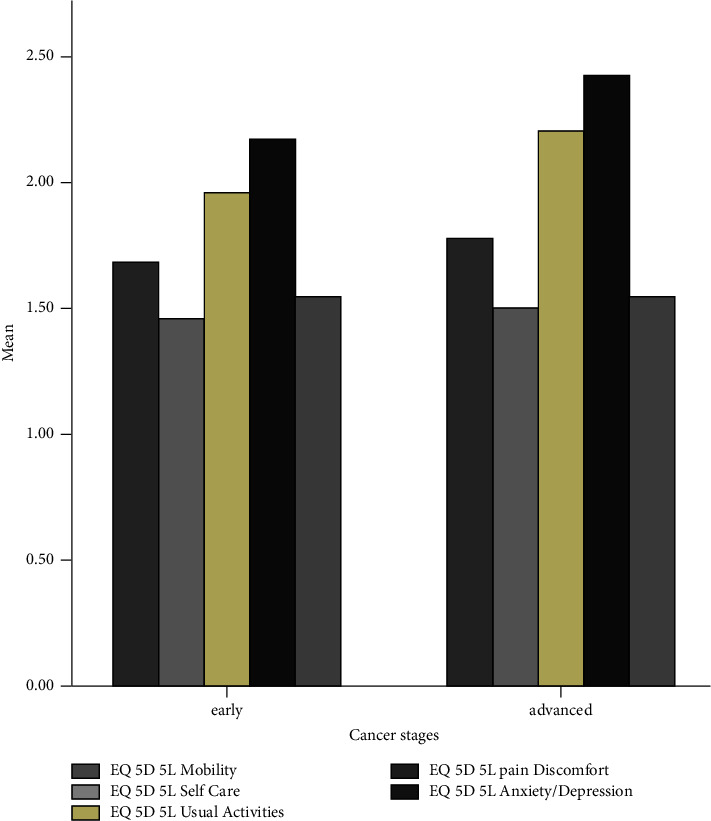
All domains of the EQ-5D-5L.

Figure 6 shows that all domains of the EQ-5D-5L have higher scales at the advanced cancer stages. This finding indicates that the symptoms are getting severe in the advanced cancer stages.

The statistical analysis discovered the factors influencing the HRQoL, as presented in Table 6. The physical and role functions are mostly influenced by the patients' characteristics. However, the VAS is only influenced by cancer stages and marital status.

Table 7 presents that the EORTC QLQ-C30 domains have significant correlations with the EQ-5D-5L domains. Meanwhile, the physical and role functions have significant correlations with mobility and daily activities. Social functions have significant correlations with usual activities. Fatigue and nausea have significant correlations with mobility and usual care. Finally, constipation and diarrhea are more correlated with self-care.

## 4. Discussion

Our study finds that the patients' characteristics could predict their HRQol. All the domains and symptoms in both questionnaires are better in the early stages of cancer than in the advanced stages. As aforementioned, the most frequently found cancer types in Indonesia are breast, cervical, and colorectal, and consecutive cancers [1]. This study has discovered that breast and colorectal cancers show the highest incidence. Other characteristics, such as low income, low education levels, no particular jobs, and young age, are significantly associated with the deterioration of the patients' HRQol. These results are consistent with those of a previous study conducted in India, which revealed that lower education, single-marital status, and higher income are associated with psychological and social domains of HRQoL [15]. Meanwhile, age, socioeconomic status, and living environment could determine cancer patients' HRQoL in Pakistan [16]. In China, economic conditions and marital status are the strong determinants of HRQoL in China [17]. The role function is the most important part of the EORTC QLQ-C30, which can be predicted by age, sex, work, and marital status. Furthermore, only the VAS of the part of the EQ-5D-5L could be determined by cancer stages and marital status.

The univariate analysis of the EORTC QLQ-C30 has discovered that some domains have a higher score (>75), denoting that the patients' HRQoL is good [5]. The symptoms of the disease that are probably caused by chemotherapy side effects also have a low score (<75) [5], indicating that the symptoms are not severe. Unlike the results of a study conducted in Ethiopia, the results of our study have a higher score of domains and a lower score of symptoms [5]. However, our findings are consistent with those of a previous study conducted in Finland which discovered that the domains have high scores, but the symptoms have low scores. Furthermore, fatigue, pain, and insomnia scores are higher in palliative patients [18]. A study conducted in Saudi Arabia also found that the domains have very high scores, and the symptoms are not severe [2]. These different results can be caused by the psychological intervention, education, or counseling provided by the health staff. The health system services also play a significant role in providing security and comfort situations during the cancer treatment [19]. All the domains of both questionnaires are lower in an advanced stage of cancer. Moreover, the EORTC QLQ-C30 symptoms get severe in the advanced stages of cancer. These findings are in line with those of a previous study conducted in Ethiopia, which have revealed that patients with advanced diseases or metastatic diseases demonstrate a worse health-related quality of life [20].

Furthermore, the EQ-5D-5L domains present that the majority of patients have no significant problems in most domains; this finding agrees with that of a study in Ethiopia [5]. The utility index in Indonesia's cancer patients is 0.68 or lower than that in Ethiopia. The EQ-5D-5L measurement has discovered that the most severe problem experienced by cancer patients is pain/discomfort. It is understandable because the pain in cancer disease is included in a palliative program and only painkillers are available to overcome the complaints. Cancer pain may cause anxiety and limit the daily activities [21]. Performing daily activities also becomes an obstacle for Indonesia's cancer patients. The limitation to move and get around may cause fatigue and influence the HRQol of the patients. Thus, routine activities must be planned [22].

We compare the score of the global health from the EORTC QLQ-C30 and the VAS of the EQ-5D-5L. We found that both of them had a score of <75, [23, 24] signifying that the patients' HRQoL has been affected [5]. Moreover, the utility value of EQ (5D) 5L is lower than that of the Indonesian general healthy population at 0.91 [25]. The score of global health status in the EORTC QLQ-C30 is lower than that of the VAS in the EQ-5D-5L. This could be caused by the instruments' characteristics; the EORTC QLQ-C30 is a specific instrument while the EQ-5D-5L is a generic instrument [26]. However, in some countries, EQ-5D-5L is validated as the instrument used in cancer patients [23, 24, 27].

The previous study, which was conducted in developing countries, used the Ordinary Least Squares to predict the models of utility of EORTC QLQ-C30 from EQ-5D-5L and SF-6D-V2 in colorectal and breast cancers. The study showed that there was a good model for predicting utility values, which were measured by nonpreference instruments [28]. Physical functions, role functions, and some symptoms in the EORTC QLQ-C30 significantly correlate with mobility and daily activities of the EQ-5D-5L. It indicates that when cancer patients are not aware of their limited activities and mobility, their physical and role functions will be affected. After showing the significant correlation among similar domains of both questionnaires, the measurement of cancer patients' HRQoL concludes that the HRQol of cancer patients is poor. We acknowledged that this study still has limitations, such as incomplete data on cancer staging and the unavailability of all medication parameters analyzed as a determinant of HRQol. These limitations could be improved by future studies.

## 5. Conclusion

This study concludes that patients' characteristics could determine cancer patients' HRQoL. The poor HRQol domains encourage health staff to provide additional care, especially related to psychosocial support. The similar domains of the EORTC QLQ-C30 and EQ-5D-5L are significantly correlated.

## Figures and Tables

**Table 1:** Transformation scoring for EORTC QLQ-C30.

Calculations	Scales
*S* = (1 − (*RS* − 1/range)) × 100	Functional scales
*S* = (*RS* − 1/range) × 100	Symptom scales
*S* = (*RS* − 1/range) × 100	Global health status

**Table 2:** Patients' characteristics.

Characteristics	*N* (451)	%
*Sex*		
Male	138	30.6
Female	313	69.4
*Age (years old)*		
25–59	388	86
≥60	63	14
*Cancer stages*		
Early	83	18.4
Advanced	253	54.1
No	115	25.5
*Education*		
No school	20	4.4
Elementary	146	32.4
Junior high school	63	14
Senior high school	135	29.9
>Senior high school	87	19.3
*Work*		
Yes	172	38.1
No	279	61.9
*Salary (IDR)*		
<2.500.000	197	43.7
≤2.500.000	254	56.3
*Marital status*		
Yes	395	87.6
No	56	12.4

IDR, Indonesian rupiah.

**Table 3:** Patients' cancer types.

Cancer diagnosed	*N* (451)	%
Breast	161	35.7
Colorectal	161	35.7
Nasopharyngeal	45	10
Lymphoma	16	3.5
Cervical	15	3.3
Lung	11	2.4
Ovarium	9	2
Others	33	7.4

**Table 4:** Domains of the European Organization for Research and Treatment for Cancer (EORTC QLQ-C30).

Domains	Average scores	SD
*Functional scales*		
Physical functions	73.72	26.23
Role functions	65.56	33.79
Emotional functions	83.13	19.51
Cognitive functions	89.43	15.98
Social functions	80.97	23.97

*Symptoms*		
Fatigue	39.42	2.52
Nausea-vomiting	25.98	2.65
Pain	36.11	3.81
Dyspnea	7.24	1.99
Insomnia	34.96	3.71
Appetite loss	33.48	3.28
Constipation	16.26	2.78
Diarrhea	7.39	1.88
Financial difficulties	32.82	3.42
Global health	68.05	20.9

**Table 5:** Patients' proportion in each domain of Europe Quality of Life 5 Dimensions 5 Level (EQ-5D-5L).

Domains	Proportion (%) in severity of problems				
	No	Slight	Moderate	Severe	Unable/extreme
Mobility	59.1	20.9	11.0	4.4	3.7
Self-care	76.3	9.9	6.2	3.1	3.7
Usual activities	42.9	28.1	13.0	5.9	9.2
Pain/discomfort	31.4	29.9	20.7	8.1	9.0
Anxiety/depression	59.6	25.5	11.5	2.4	0.2

**Table 6:** The patients' characteristics related to the EORTC QLQ-C30 and EQ-5D-5L domains.

Patients' characteristics	EORTC QLQ-C30 (*p* values)	EQ-5D-5L (*p* values)
Cancer stages	Physical functions (0.01)^*∗*^	VAS (0.00)^*∗*^
	Social functions (0.18)	

Age	Role functions (0.028)^*∗*^	

Sex	Role functions (0.003)^*∗*^	
	Global health (0.04)^*∗*^	

Work	Role functions (0.04)^*∗*^	
	Dyspnea (0.003)^*∗*^	

Marital status	Role functions (0.002)^*∗*^	VAS (0.004)^*∗*^
	Dyspnea (0.028)^*∗*^	
	Global health (0.007)^*∗*^	

Salary	Physical functions (0.10)	
	Emotional functions (0.04)^*∗*^	
	Cognitive functions (0.07)^*∗*^	
	Social functions (0.14)	

VAS: visual analog scale and ^*∗*^statistically significant (*p* value<0.05).

**Table 7:** Correlations between EORTC QLQ-C30 and EQ-5D-5L domains.

	Mobility	Self-care	Usual activities	Pain/discomfort	Anxiety/depression
Physical functions	**0.729**	**0.668**	**0.751**	0.523	0.22
Role functions	**0.579**	0.472	**0.672**	0.502	0.223
Pain	0.487	0.356	0.555	**0.836**	0.256
Emotional functions	0.303	0.291	0.282	0.163	**0.444**
Social functions	0.353	0.319	**0.538**	0.393	0.303
Fatigue	**0.434**	0.379	**0.497**	0.406	0.306
Nausea	**0.142**	0.071	**0.246**	0.061	0.123
Constipation and diarrhea	0.173	**0.200**	0.170	0.171	0.148

The domain numbers presented are the correlation coefficients. Numbers written in bold mean that the correlations are statistically significant (*p* value <0.05).

## Data Availability

The data used in this study are made available upon reasonable request to the corresponding author.

## Supplementary Materials

Supplementary Materials The supplementary data are questionnaire form and the data sheet of this study.
