# Supplementary material for: Quality of Life of Cancer Patients during Chemotherapy in Indonesia: A Comparison of EORTC QLQ-C30 and EQ-5D-5L, Based on Patients' Characteristics
Source: Int J Clin Pract. 2023 Mar 3;2023:9357299. doi: 10.1155/2023/9357299 (PMC10005864; doi:10.1155/2023/9357299)
Supplement: Supplementary Materials — The supplementary data are questionnaire form and the data sheet of this study. [file 9357299.f1.pdf]

Jenis Kelamin (Sex);		Usia (tahun);	Age (ye	Diagnosa		Stadium Kanker (canc		Biaya (Health insuran	
P	2	36	1	Ca Mamm	1	2	1	BPJS	1
P	2	47	1	Ca Mamm	1	3	2	BPJS	1
P	2	46	1	Ca Mamm	1	2	1	BPJS	1
P	2	45	1	Ca Mamm	1	4	2	BPJS	1
P	2	47	1	Ca Mamm	1	2	1	BPJS	1
P	2	45	1	Ca Mamm	1	3	2	BPJS	1
P	2	43	1	Ca Mamm	1	3	2	BPJS	1
P	2	54	1	Ca Mamm	1	4	2	BPJS	1
P	2	47	1	Ca Mamm	1	1	1	BPJS	1
P	2	37	1	Ca Mamm	1	3	2	BPJS	1
P	2	50	1	Ca Mamm	1	2	1	BPJS	1
P	2	57	1	Ca Mamm	1	3	2	BPJS	1
P	2	30	1	Ca Mamm	1	2	1	BPJS	1
P	2	32	1	Ca Mamm	1	3	2	BPJS	1
P	2	60	1	Ca Mamm	1	2	1	BPJS	1
P	2	58	1	Ca Mamm	1	2	1	BPJS	1
P	2	41	1	Ca Mamm	1	3	2	BPJS	1
P	2	45	1	Ca Mamm	1	2	1	BPJS	1
P	2	42	1	Ca Mamm	1	4	2	BPJS	1
P	2	50	1	Ca Mamm	1	2	1	BPJS	1
P	2	37	1	Ca Mamm	1	3	2	BPJS	1
P	2	44	1	Ca Mamm	1	2	1	BPJS	1
P	2	64	2	Ca Mamm	1	2	1	BPJS	1
P	2	46	1	Ca Mamm	1	2	1	BPJS	1
P	2	57	1	Ca Mamm	1	2	1	BPJS	1
P	2	53	1	Ca Mamm	1	3	2	BPJS	1
P	2	58	1	Ca Mamm	1	2	1	BPJS	1
P	2	39	1	Ca Mamm	1	4	2	BPJS	1
P	2	40	1	Ca Mamm	1	4	2	BPJS	1
P	2	46	1	Ca Mamm	1	4	2	BPJS	1
P	2	43	1	Ca Mamm	1	4	2	BPJS	1
P	2	67	2	Ca Mamm	1	2	1	BPJS	1
P	2	47	1	Ca Mamm	1	2	1	BPJS	1
P	2	39	1	Ca Mamm	1	4	2	BPJS	1
P	2	73	2	Ca Mamm	1	3	2	BPJS	1
P	2	42	1	Ca Mamm	1	4	2	BPJS	1
P	2	54	1	Ca Mamm	1	2	1	BPJS	1
P	2	40	1	Ca Mamm	1	2	1	BPJS	1
P	2	50	1	Ca Mamm	1	4	2	BPJS	1
P	2	40	1	Ca Mamm	1	1	1	BPJS	1
P	2	56	1	Ca Mamm	1	3	2	BPJS	1
P	2	48	1	Ca Mamm	1	1	1	BPJS	1
P	2	49	1	Ca Mamm	1	4	2	BPJS	1
P	2	46	1	Ca Mamm	1	3	2	BPJS	1
P	2	35	1	Ca Mamm	1	2	1	BPJS	1
P	2	42	1	Ca Mamm	1	2	1	BPJS	1

P	2	47	1	Ca Mamm	1	2	1	BPJS	1
P	2	39	1	Ca Mamm	1	2	1	BPJS	1
P	2	55	1	Ca Mamm	1	2	1	BPJS	1
P	2	52	1	Ca Mamm	1	4	2	BPJS	1
P	2	36	1	Ca Mamm	1	3	2	BPJS	1
P	2	31	1	Ca Mamm	1	3	2	BPJS	1
P	2	42	1	Ca Mamm	1	2	1	BPJS	1
P	2	52	1	Ca Mamm	1	2	1	BPJS	1
P	2	54	1	Ca Mamm	1	3	2	BPJS	1
P	2	50	1	Ca Mamm	1	2	1	BPJS	1
P	2	30	1	Ca Mamm	1	2	1	BPJS	1
P	2	49	1	Ca Mamm	1	2	1	BPJS	1
P	2	52	1	Ca Mamm	1	3	2	BPJS	1
P	2	49	1	Ca Mamm	1	4	2	BPJS	1
P	2	32	1	Ca Mamm	1	2	1	BPJS	1
P	2	54	1	Ca Mamm	1	4	2	BPJS	1
P	2	31	1	Ca Mamm	1	2	1	BPJS	1
P	2	52	1	Ca Mamm	1	3	2	BPJS	1
P	2	61	2	Ca Mamm	1	2	1	BPJS	1
L	1	52	1	Ca Nasofa	2	4	2	BPJS	1
L	1	20	1	Ca Nasofa	2	4	2	BPJS	1
P	2	44	1	Ca Nasofa	2	3	2	BPJS	1
L	1	68	2	Ca Nasofa	2	4	2	BPJS	1
L	1	32	1	Ca Nasofa	2	4	2	BPJS	1
L	1	35	1	Ca Nasofa	2	3	2	BPJS	1
L	1	60	1	Ca Nasofa	2	3	2	BPJS	1
P	2	50	1	Ca Nasofa	2	3	2	BPJS	1
L	1	51	1	Ca Nasofa	2	4	2	BPJS	1
L	1	39	1	Ca Nasofa	2	3	2	BPJS	1
L	1	51	1	Ca Nasofa	2	4	2	BPJS	1
P	2	54	1	Ca Nasofa	2	4	2	BPJS	1
P	2	57	1	Ca Nasofa	2	4	2	BPJS	1
L	1	44	1	Ca Nasofa	2	4	2	BPJS	1
P	2	42	1	Ca Nasofa	2	4	2	BPJS	1
L	1	54	1	Ca Nasofa	2	3	2	BPJS	1
P	2	46	1	Ca Nasofa	2	3	2	BPJS	1
L	1	60	1	Ca Nasofa	2	4	2	BPJS	1
L	1	58	1	Ca Nasofa	2	4	2	BPJS	1
P	2	24	1	Ca Nasofa	2	3	2	BPJS	1
P	2	45	1	Ca Colorec	3	4	2	BPJS	1
L	1	62	2	Ca Colorec	3	4	2	BPJS	1
P	2	50	1	Ca Colorec	3	3	2	BPJS	1
P	2	47	1	Ca Colorec	3	3	2	BPJS	1
P	2	62	2	Ca Colorec	3	4	2	BPJS	1
P	2	40	1	Ca Colorec	3	4	2	BPJS	1
L	1	52	1	Ca Colorec	3	4	2	BPJS	1
L	1	60	1	Ca Colorec	3	4	2	BPJS	1

L	1	64	2	Ca Colore	3	4	2	BPJS	1
P	2	51	1	Ca Colore	3	4	2	BPJS	1
P	2	63	2	Ca Colore	3	4	2	BPJS	1
L	1	56	1	Ca Colore	3	4	2	BPJS	1
L	1	21	1	Ca Colore	3	3	2	BPJS	1
P	2	26	1	Ca Colore	3	3	2	BPJS	1
P	2	53	1	Ca Colore	3	4	2	BPJS	1
L	1	61	2	Ca Colore	3	2	1	BPJS	1
P	2	59	1	Ca Colore	3	4	2	BPJS	1
L	1	51	1	Ca Colore	3	4	2	BPJS	1
L	1	50	1	Ca Colore	3	3	2	BPJS	1
L	1	48	1	Ca Colore	3	4	2	BPJS	1
P	2	55	1	Ca Colore	3	2	1	BPJS	1
P	2	63	2	Ca Colore	3	4	2	BPJS	1
P	2	55	1	Ca Colore	3	3	2	BPJS	1
L	1	43	1	Ca Colore	3	4	2	BPJS	1
P	2	47	1	Ca Colore	3	3	2	BPJS	1
P	2	66	2	Ca Colore	3	3	2	BPJS	1
P	2	52	1	Ca Colore	3	3	2	BPJS	1
L	1	31	1	Ca Colore	3	4	2	BPJS	1
L	1	48	1	Ca Colore	3	4	2	BPJS	1
P	2	55	1	Ca Colore	3	3	2	BPJS	1
P	2	39	1	Ca Colore	3	4	2	BPJS	1
P	2	26	1	Ca Colore	3	3	2	BPJS	1
L	1	26	1	Ca Colore	3	4	2	BPJS	1
L	1	45	1	Ca Colore	3	4	2	BPJS	1
L	1	54	1	Ca Colore	3	4	2	BPJS	1
L	1	50	1	Ca Colore	3	4	2	BPJS	1
P	2	51	1	Ca Colore	3	4	2	BPJS	1
P	2	49	1	Ca Colore	3	3	2	BPJS	1
L	1	48	1	Ca Colore	3	4	2	BPJS	1
L	1	52	1	Ca Colore	3	4	2	BPJS	1
L	1	39	1	Ca Colore	3	4	2	BPJS	1
L	1	42	1	Ca Colore	3	4	2	BPJS	1
P	2	52	1	Ca Colore	3	3	2	BPJS	1
L	1	46	1	Ca Colore	3	4	2	BPJS	1
L	1	45	1	Ca Colore	3	3	2	BPJS	1
P	2	34	1	Ca Colore	3	3	2	BPJS	1
L	1	45	1	Ca Colore	3	4	2	BPJS	1
L	1	31	1	Ca Colore	3	4	2	BPJS	1
P	2	39	1	Ca Colore	3	3	2	BPJS	1
L	1	54	1	Ca Colore	3	4	2	BPJS	1
L	1	45	1	Ca Colore	3	4	2	BPJS	1
L	1	31	1	Ca Colore	3	4	2	BPJS	1
P	2	36	1	Ca Colore	3	4	2	BPJS	1
P	2	39	1	Ca Colore	3	3	2	BPJS	1
P	2	38	1	Ca Colore	3	4	2	BPJS	1

L	1	37	1	Ca Colore	3	4	2	BPJS	1
L	1	55	1	Ca Colore	3	3	2	BPJS	1
P	2	55	1	Ca Mamm	1	2	1	BPJS	1
P	2	52	1	Ca Mamm	1	3	2	BPJS	1
P	2	59	1	Ca Mamm	1	2	1	BPJS	1
P	2	32	1	Ca Mamm	1	2	1	BPJS	1
P	2	55	1	Ca Mamm	1	4	2	BPJS	1
P	2	37	1	Ca Mamm	1	3	2	BPJS	1
P	2	46	1	Ca Mamm	1	3	2	BPJS	1
L	1	54	1	Ca Colore	3	3	2	BPJS	1
P	2	58	1	Ca Mamm	1	2	1	BPJS	1
L	1	39	1	Ca Colore	3	4	2	BPJS	1
P	2	59	1	Ca Mamm	1	2	1	Umum	2
L	1	49	1	Ca Colore	3	3	2	BPJS	1
P	2	59	1	Ca Colore	3	4	2	BPJS	1
L	1	67	2	Ca Colore	3	4	2	BPJS	1
P	2	63	2	Ca Colore	3	4	2	BPJS	1
L	1	53	1	Ca Nasofa	2	4	2	BPJS	1
P	2	41	1	Ca Mamm	1	4	2	BPJS	1
P	2	46	1	Ca Colore	3	2	1	BPJS	1
P	2	45	1	Ca Mamm	1	3	2	BPJS	1
P	2	38	1	Ca Mamm	1	2	1	BPJS	1
P	2	36	1	Ca Colore	3	4	2	BPJS	1
L	1	49	1	Ca Colore	3	2	1	BPJS	1
P	2	37	1	Ca Mamm	1	2	1	BPJS	1
P	2	46	1	Ca Colore	3	4	2	Umum	2
P	2	61	2	Ca Mamm	1	2	1	BPJS	1
L	1	45	1	Ca Nasofa	2	4	2	BPJS	1
L	1	39	1	Ca Nasofa	2	4	2	BPJS	1
P	2	63	2	Ca Mamm	1	2	1	BPJS	1
P	2	47	1	Ca Nasofa	2	3	2	BPJS	1
P	2	55	1	Ca Mamm	1	4	2	BPJS	1
P	2	61	2	Ca Colore	3	4	2	BPJS	1
P	2	38	1	Ca Mamm	1	4	2	BPJS	1
L	1	41	1	Ca Nasofa	3	4	2	BPJS	1
P	2	61	2	Ca Nasofa	3	3	2	BPJS	1
P	2	55	1	Ca Colore	3	4	2	BPJS	1
L	1	69	2	Ca Nasofa	2	4	2	BPJS	1
P	2	32	1	Ca Mamm	1	3	2	BPJS	1
P	2	36	1	Ca Mamm	1	2	1	BPJS	1
L	1	58	1	Ca Colore	3	4	2	BPJS	1
P	2	55	1	Ca Colore	3	4	2	BPJS	1
L	1	61	2	Ca Nasofa	2	4	2	BPJS	1
P	2	24	1	Ca Colore	3	4	2	BPJS	1
P	2	52	1	Ca Mamm	1	2	1	BPJS	1
P	2	59	1	Ca Mamm	1	4	2	BPJS	1
L	1	22	1	Ca Colore	3	4	2	BPJS	1

P	2	59	1	Ca Mamm	1	2	1	BPJS	1
P	2	49	1	Ca Nasofa	2	4	2	BPJS	1
P	2	47	1	Ca Colore	3	4	2	BPJS	1
P	2	53	1	Ca Mamm	1	2	1	BPJS	1
P	2	59	1	Ca Mamm	1	4	2	BPJS	1
P	2	62	2	Ca Mamm	1	3	2	BPJS	1
L	1	26	1	Ca Nasofa	2	4	2	BPJS	1
P	2	50	1	Ca Colore	3	3	2	BPJS	1
P	2	59	1	Ca Colore	3	4	2	BPJS	1
P	2	36	1	Ca Colore	3	4	2	BPJS	1
L	1	48	1	Ca Nasofa	2	4	2	BPJS	1
P	2	59	1	Ca Colore	3	4	2	BPJS	1
P	2	60	1	Ca Colore	3	4	2	BPJS	1
P	2	38	1	Ca Mamm	1	3	2	BPJS	1
P	2	41	1	Ca Mamm	1	3	2	BPJS	1
P	2	55	1	Ca Mamm	1	2	1	BPJS	1
P	2	60	1	Ca Colore	3	4	2	BPJS	1
P	2	22	1	Ca Colore	3	4	2	BPJS	1
P	2	64	2	Ca Colore	3	4	2	BPJS	1
L	1	34	1	Ca Colore	3	4	2	BPJS	1
P	2	37	1	Ca Mamm	1	2	1	BPJS	1
P	2	55	1	Ca Mamm	1	2	1	BPJS	1
L	1	61	2	Ca Nasofa	2	4	2	BPJS	1
P	2	35	1	Ca Mamm	1	3	2	BPJS	1
P	2	33	1	Ca Mamm	1	4	2	BPJS	1
P	2	60	1	Ca Mamm	1	4	2	BPJS	1
P	2	40	1	Ca Mamm	1	4	2	BPJS	1
P	2	54	1	Ca Colore	3	3	2	BPJS	1
P	2	66	2	Ca Mamm	1	4	2	BPJS	1
P	2	50	1	Ca Mamm	1	4	2	BPJS	1
P	2	40	1	Ca Mamm	1	4	2	BPJS	1
P	2	42	1	Ca Nasofa	2	4	2	BPJS	1
P	2	34	1	Ca Mamm	1	4	2	BPJS	1
P	2	53	1	Ca Mamm	1	2	1	BPJS	1
P	2	57	1	Ca Mamm	1	3	2	BPJS	1
P	2	57	1	Ca Mamm	1	2	1	BPJS	1
L	1	33	1	Ca Nasofa	2	3	2	BPJS	1
L	1	49	1	Ca Colore	3	3	2	BPJS	1
P	2	50	1	Ca Mamm	1	2	1	BPJS	1
P	2	66	2	Ca Colore	3	3	2	BPJS	1
P	2	50	1	Ca Colore	3	4	2	BPJS	1
P	2	33	1	Ca Mamm	1	2	1	BPJS	1
L	1	56	1	Ca Colore	3	4	2	BPJS	1
L	1	21	1	Ca Colore	3	3	2	BPJS	1
P	2	65	2	Ca Mamm	1	4	2	BPJS	1
L	1	44	1	Ca Nasofa	2	4	2	BPJS	1
L	1	58	1	Ca Colore	3	4	2	BPJS	1

L	1	53	1	Ca Colorec	3	4	2	BPJS	1
L	1	60	1	Ca Colorec	3	4	2	BPJS	1
L	1	51	1	Ca Nasofa	2	4	2	BPJS	1
P	2	60	1	Ca Colorec	3	4	2	BPJS	1
P	2	67	2	Ca Colorec	3	4	2	BPJS	1
L	1	57	1	Ca Colorec	3	3	2	BPJS	1
L	1	48	1	Ca Colorec	3	4	2	BPJS	1
L	1	37	1	Ca Colorec	3	3	2	BPJS	1
P	2	48	1	Ca Nasofa	2	4	2	BPJS	1
P	2	39	1	Ca Mamm	1	3	2	BPJS	1
P	2	51	1	Ca Mamm	1	4	2	BPJS	1
P	2	58	1	Ca Mamm	1	4	2	BPJS	1
P	2	67	2	Ca Mamm	1	4	2	BPJS	1
P	2	42	1	Ca Nasofa	2	4	2	BPJS	1
P	2	56	1	Ca Mamm	1	3	2	BPJS	1
P	2	24	1	Ca Colorec	3	4	2	BPJS	1
L	1	68	2	Ca Colorec	3	4	2	BPJS	1
L	1	56	1	Ca Colorec	3	4	2	BPJS	1
P	2	37	1	Ca Mamm	1	4	2	BPJS	1
P	2	53	1	Ca Mamm	1	2	1	BPJS	1
P	2	54	1	Ca Mamm	1	4	2	BPJS	1
P	2	53	1	Ca Mamm	1	3	2	BPJS	1
P	2	41	1	Ca Mamm	1	4	2	BPJS	1
L	1	41	1	Ca Colorec	3	3	2	BPJS	1
P	2	51	1	Ca Mamm	1	2	1	BPJS	1
P	2	45	1	Ca Colorec	3	4	2	BPJS	1
P	2	65	2	Ca Colorec	3	4	2	BPJS	1
P	2	48	1	Ca Colorec	3	3	2	BPJS	1
P	2	36	1	Ca Mamm	1	3	2	BPJS	1
P	2	36	1	Ca Colorec	3	4	2	BPJS	1
P	2	58	1	Ca Colorec	3	3	2	BPJS	1
P	2	56	1	Ca Mamm	1	4	2	BPJS	1
P	2	41	1	Ca Mamm	1	2	1	BPJS	1
P	2	48	1	Ca Mamm	1	2	1	BPJS	1
P	2	61	2	Ca Mamm	1	3	2	BPJS	1
P	2	57	1	Ca Mamm	1	3	2	BPJS	1
P	2	51	1	Ca Mamm	1	2	1	BPJS	1
P	2	51	1	Ca Mamm	1	4	2	BPJS	1
P	2	54	1	Ca Mamm	1	4	2	BPJS	1
L	1	29	1	Ca Colorec	3	3	2	BPJS	1
P	2	48	1	Ca Colorec	3	3	2	BPJS	1
P	2	50	1	Ca Mamm	1	3	2	BPJS	1
P	2	55	1	Ca Colorec	3	4	2	BPJS	1
L	1	35	1	Ca Colorec	3	3	2	BPJS	1
P	2	54	1	Ca Colorec	3	4	2	BPJS	1
P	2	29	1	Ca Mamm	1	2	1	BPJS	1
L	1	25	1	Ca Colorec	3	3	2	BPJS	1

L	1	30	1	Ca Nasofa	2	3	2	BPJS	1
P	2	60	1	Ca Mamm	1	1	1	BPJS	1
P	2	58	1	Ca Mamm	1	4	2	BPJS	1
P	2	61	2	Ca Nasofa	2	4	2	BPJS	1
P	2	54	1	Ca Colorec	3	4	2	BPJS	1
P	2	38	1	Ca Mamm	1	4	2	BPJS	1
P	2	51	1	Ca Mamm	1	4	2	BPJS	1
P	2	47	1	Ca Mamm	1	3	2	BPJS	1
L	1	49	1	Ca Nasofa	2	3	2	BPJS	1
P	2	65	2	Ca Colorec	3	3	2	BPJS	1
P	2	43	1	Ca Colorec	3	4	2	BPJS	1
P	2	51	1	Ca Colorec	3	3	2	BPJS	1
L	1	62	2	Ca Colorec	3	4	2	BPJS	1
L	1	40	1	Ca Colorec	3	4	2	BPJS	1
P	2	46	1	Ca Mamm	1	3	2	BPJS	1
L	1	54	1	Ca Colorec	3	3	2	BPJS	1
P	2	53	1	Ca Mamm	1	4	2	BPJS	1
P	2	54	1	Ca Mamm	1	2	1	BPJS	1
L	1	21	1	Ca Colorec	3	3	2	BPJS	1
L	1	58	1	Ca Paru	4	3	2	BPJS	1
L	1	43	1	Ca Paru	4	4	2	BPJS	1
L	1	62	2	Ca Paru	4	4	2	BPJS	1
L	1	50	1	Ca Paru	4	0	0	BPJS	1
L	1	49	1	Ca Paru	4	0	0	BPJS	1
L	1	49	1	Ca Paru	4	4	2	BPJS	1
P	2	66	2	Ca Paru	4	0	0	BPJS	1
P	2	49	1	Ca Paru	4	0	0	BPJS	1
L	1	58	1	Ca Paru	4	0	0	BPJS	1
L	1	64	2	Ca Paru	4	0	0	BPJS	1
L	1	41	1	Ca Paru	4	0	0	BPJS	1
P	2	56	1	Ca Limfor	5	0	0	BPJS	1
L	1	61	2	Ca Limfor	5	0	0	BPJS	1
L	1	17	1	Ca Limfor	5	4	2	BPJS	1
L	1	66	2	Ca Limfor	5	0	0	BPJS	1
L	1	49	1	Ca Limfor	5	0	0	BPJS	1
P	2	21	1	Ca Limfor	5	0	0	BPJS	1
P	2	36	1	Ca Limfor	5	0	0	BPJS	1
P	2	52	1	Ca Limfor	5	0	0	BPJS	1
L	1	48	1	Ca Limfor	5	0	0	BPJS	1
P	2	57	1	Ca Limfor	5	0	0	BPJS	1
L	1	64	2	Ca Limfor	5	0	0	BPJS	1
P	2	50	1	Ca Limfor	5	0	0	BPJS	1
P	2	56	1	Ca Limfor	5	0	0	BPJS	1
L	1	44	1	Ca Limfor	5	0	0	BPJS	1
L	1	56	1	Ca Limfor	5	0	0	BPJS	1
L	1	68	2	Ca Limfor	5	0	0	BPJS	1
P	2	52	1	Ca Ovariu	6	0	0	BPJS	1

P	2	28	1	Ca Ovariu	6	3	2	BPJS	1
P	2	58	1	Ca Ovariu	6	3	2	BPJS	1
P	2	53	1	Ca Ovariu	6	0	0	BPJS	1
P	2	52	1	Ca Ovariu	6	0	0	BPJS	1
P	2	47	1	Ca Ovariu	6	2	1	BPJS	1
P	2	55	1	Ca Ovariu	6	3	2	BPJS	1
P	2	45	1	Ca Ovariu	6	3	2	BPJS	1
P	2	55	1	Ca Ovariu	6	3	2	BPJS	1
P	2	42	1	Ca Endor	7	0	0	BPJS	1
P	2	54	1	Ca Endor	7	3	2	BPJS	1
P	2	34	1	Ca Fibrob	8	0	0	BPJS	1
P	2	46	1	Ca Fibrob	8	0	0	BPJS	1
P	2	22	1	Ca Fibrob	8	0	0	BPJS	1
P	2		1	Ca Fibrob	8	0	0	BPJS	1
P	2	55	1	Ca Fibrob	8	0	0	KIS	1
P	2	54	1	Ca Fibrob	8	0	0	BPJS	1
P	2	31	1	Ca Fibrob	8	0	0	BPJS	1
P	2	55	1	Ca Axilla	9	0	0	BPJS	1
L	1	52	1	Ca Laring	10	0	0	BPJS	1
L	1	60	1	Ca Bulli	11	0	0	BPJS	1
L	1	62	2	Ca Bulli	11	0	0	BPJS	1
L	1	63	2	Ca Bulli	11	0	0	BPJS	1
L	1	62	2	Ca Bulli	11	0	0	BPJS	1
P	2	58	1	Ca Urothe	12	0	0	BPJS	1
L	1	65	2	Ca Nasofa	2	0	0	BPJS	1
L	1	54	1	Ca Nasofa	2	0	0	BPJS	1
P	2	46	1	Ca Nasofa	2	0	0	BPJS	1
L	1	41	1	Ca Nasofa	2	0	0	BPJS	1
L	1	61	2	Ca Nasofa	2	0	0	BPJS	1
L	1	25	1	Ca Nasofa	2	0	0	BPJS	1
L	1	51	1	Ca Kulit	13	0	0	BPJS	1
L	1	67	2	Ca Kulit	13	0	0	BPJS	1
L	1	51	1	Ca Kulit	13	0	0	BPJS	1
L	1	43	1	Ca Mandi	14	1	1	BPJS	1
L	1	59	1	Ca Omen	15	0	2	BPJS	1
L	1	34	1	Ca Sarko	16	0	0	BPJS	1
P	2	52	1	Ca Sinona	17	0	0	BPJS	1
P	2	46	1	Ca Sinona	17	0	0	BPJS	1
L	1	62	2	Ca Sinona	17	0	0	BPJS	1
L	1	66	2	Ca Lidah	18	0	0	BPJS	1
L	1	72	2	Ca Colli	19	0	0	BPJS	1
P	2	49	1	Ca Colli	19	0	0	BPJS	1
P	2	54	1	Ca Meser	20	0	0	BPJS	1
L	1	38	1	Ca Testis	21	0	0	BPJS	1
P	2	50	1	Ca Lemak	22	0	0	BPJS	1
P	2	35	1	Ca Jejenu	23	0	0	BPJS	1
P	2	52	1	Ca Palatu	24	0	0	BPJS	1

L	1	65	2	Ca Colore	3	0	0	BPJS	1
P	2		1	Ca Colore	3	0	0	BPJS	1
P	2	59	1	Ca Colon	25	0	0	BPJS	1
L	1	72	2	Ca Colon	25	4	2	BPJS	1
P	2	25	1	Ca Colon	25	0	0	BPJS	1
L	1	37	1	Ca Colon	25	0	0	BPJS	1
L	1	44	1	Ca Colon	25	0	0	BPJS	1
P	2	65	2	Ca Colon	25	0	0	BPJS	1
P	2	47	1	Ca Colon	25	0	0	BPJS	1
L	1	69	2	Ca Colon	25	0	0	BPJS	1
L	1	65	2	Ca Colon	25	0	0	BPJS	1
P	2	40	1	Ca Colon	25	0	0	BPJS	1
P	2	51	1	Ca Colon	25	0	0	BPJS	1
P	2	53	1	Ca Colon	25	0	0	BPJS	1
P	2	55	1	Ca Colon	25	0	0	BPJS	1
L	1	41	1	Ca Colon	25	0	0	BPJS	1
L	1	49	1	Ca Colon	25	0	0	BPJS	1
P	2	44	1	Ca Colon	25	0	0	BPJS	1
L	1	49	1	Ca Colon	25	0	0	BPJS	1
P	2	43	1	Ca Colon	25	0	0	BPJS	1
L	1	44	1	Ca Colon	25	0	0	BPJS	1
P	2	64	2	Ca Colon	25	0	0	BPJS	1
P	2	48	1	Ca Rektu	26	0	0	BPJS	1
L	1	71	2	Ca Rektu	26	0	0	BPJS	1
P	2	31	1	Ca Rektu	26	0	0	BPJS	1
L	1	53	1	Ca Rektu	26	0	0	BPJS	1
P	2	46	1	Ca Rektu	26	0	0	BPJS	1
L	1	44	1	Ca Rektu	26	0	0	BPJS	1
P	2	57	1	Ca Rektu	26	0	0	BPJS	1
P	2	44	1	Ca Rektu	26	0	0	BPJS	1
P	2	64	2	Ca Rektu	26	0	0	BPJS	1
L	1	63	2	Ca Rektu	26	0	0	BPJS	1
P	2	50	1	Ca Rektu	26	0	0	BPJS	1
P	2	42	1	Ca Rektu	26	0	0	BPJS	1
L	1	35	1	Ca Rektu	26	0	0	BPJS	1
L	1	40	1	Ca Rektu	26	0	0	BPJS	1
P	2	64	2	Ca Rektu	26	0	0	BPJS	1
P	2	58	1	Ca Rektu	26	0	0	BPJS	1
P	2	66	2	Ca Rektu	26	0	0	BPJS	1
P	2	42	1	Ca Mamn	1	0	0	BPJS	1
P	2	49	1	Ca Mamn	1	0	0	BPJS	1
P	2	38	1	Ca Mamn	1	0	0	BPJS	1
P	2	73	2	Ca Mamn	1	2	1	BPJS	1
P	2	43	1	Ca Mamn	1	3	2	BPJS	1
P	2	43	1	Ca Mamn	1	0	0	BPJS	1
P	2	56	1	Ca Mamn	1	1	1	BPJS	1

P	2	56	1	Ca Mamn	1	3	2	BPJS	1
P	2	37	1	Ca Mamn	1	4	2	BPJS	1
P	2	54	1	Ca Mamn	1	2	1	BPJS	1
P	2	53	1	Ca Mamn	1	0	0	BPJS	1
P	2	42	1	Ca Mamn	1	0	0	BPJS	1
P	2	33	1	Ca Mamn	1	2	1	BPJS	1
P	2	55	1	Ca Mamn	1	2	1	BPJS	1
P	2	54	1	Ca Mamn	1	3	2	BPJS	1
P	2	48	1	Ca Mamn	1	0	0	BPJS	1
P	2	63	2	Ca Mamn	1	0	0	BPJS	1
P	2	60	1	Ca Mamn	1	0	0	BPJS	1
P	2	55	1	Ca Mamn	1	0	0	BPJS	1
P	2	60	1	Ca Mamn	1	0	0	BPJS	1
P	2	57	1	Ca Mamn	1	0	0	BPJS	1
P	2	47	1	Ca Mamn	1	0	0	BPJS	1
P	2	44	1	Ca Servik	27	2	1	BPJS	1
P	2	60	1	Ca Servik	27	0	0	BPJS	1
P	2	48	1	Ca Servik	27	2	1	BPJS	1
P	2	51	1	Ca Servik	27	2	1	BPJS	1
P	2	54	1	Ca Servik	27	3	2	BPJS	1
P	2	45	1	Ca Servik	27	2	1	BPJS	1
P	2	57	1	Ca Servik	27	2	1	BPJS	1
P	2	40	1	Ca Servik	27	2	1	BPJS	1
P	2	44	1	Ca Servik	27	0	0	BPJS	1
P	2	39	1	Ca Servik	27	2	1	BPJS	1
P	2	48	1	Ca Servik	27	3	2	BPJS	1
P	2	41	1	Ca Servik	27	2	1	BPJS	1
P	2	48	1	Ca Servik	27	0	0	BPJS	1
P	2	24	1	Ca Servik	27	2	1	BPJS	1
P	2	57	1	Ca Servik	27	2	1	BPJS	1

Pendidikan (educatio		Pekerjaan (work); bek		Status Pernikahan (ma		Pendapatan (Salary in IDR)	
SMU	4	Tidak Bek	2	Menikah	1	<Rp 2.500 a	1
SMU	4	Bekerja	1	Menikah	1	>RP 2.500 b	2
SMU	4	Bekerja	1	Menikah	1	<Rp 2.500 a	1
SMU	4	Bekerja	1	Menikah	1	>RP 2.500 b	2
SLTP	3	Bekerja	1	Menikah	1	<Rp 2.500 a	1
SMU	4	Tidak Bek	2	Menikah	1	<Rp 2.500 a	1
SLTP	3	Bekerja	1	Menikah	1	<Rp 2.500 a	1
SD	2	Tidak Bek	2	Menikah	1	<Rp 2.500 a	1
SMU	4	Tidak Bek	2	Menikah	1	<Rp 2.500 a	1
SLTP	3	Tidak Bek	2	Menikah	1	<Rp 2.500 a	1
SD	2	Bekerja	1	Menikah	1	<Rp 2.500 a	1
Tidak Sek	1	Bekerja	1	Menikah	1	<Rp 2.500 a	1
S1-S3	5	Bekerja	1	Tidak Mer	2	>RP 2.500 b	2
SLTP	3	Tidak Bek	2	Menikah	1	<Rp 2.500 a	1
S1-S3	5	Bekerja	1	Tidak Mer	2	>RP 2.500 b	2
D3	5	Bekerja	1	Menikah	1	>RP 2.500 b	2
SMU	4	Tidak Bek	2	Menikah	1	<Rp 2.500 a	1
S1-S3	5	Bekerja	1	Menikah	1	>RP 2.500 b	2
SLTP	3	Tidak Bek	2	Menikah	1	<Rp 2.500 a	1
SMU	4	Tidak Bek	2	Menikah	1	<Rp 2.500 a	1
SD	2	Bekerja	1	Menikah	1	<Rp 2.500 a	1
SLTP	3	Bekerja	1	Menikah	1	<Rp 2.500 a	1
SMU	4	Bekerja	1	Menikah	1	>RP 2.500 b	2
SLTP	3	Bekerja	1	Menikah	1	>RP 2.500 b	2
S1-S3	5	Bekerja	1	Menikah	1	>RP 2.500 b	2
S1-S3	5	Bekerja	1	Menikah	1	>RP 2.500 b	2
S1-S3	5	Bekerja	1	Menikah	1	>RP 2.500 b	2
S1-S3	5	Bekerja	1	Tidak Mer	2	<Rp 2.500 a	1
SD	2	Tidak Bek	2	Menikah	1	<Rp 2.500 a	1
SLTP	3	Tidak Bek	2	Tidak Mer	2	<Rp 2.500 a	1
SLTP	3	Tidak Bek	2	Tidak Mer	2	<Rp 2.500 a	1
S1-S3	5	Bekerja	1	Menikah	1	>RP 2.500 b	2
SD	2	Tidak Bek	2	Menikah	1	>RP 2.500 b	2
SMU	4	Bekerja	1	Tidak Mer	2	<Rp 2.500 a	1
SMU	4	Tidak Bek	2	Tidak Mer	2	<Rp 2.500 a	1
SLTP	3	Tidak Bek	2	Menikah	1	<Rp 2.500 a	1
SD	2	Bekerja	1	Menikah	1	<Rp 2.500 a	1
SD	2	Bekerja	1	Tidak Mer	2	<Rp 2.500 a	1
S1-S3	5	Bekerja	1	Menikah	1	>RP 2.500 b	2
SMU	4	Bekerja	1	Menikah	1	<Rp 2.500 a	1
SMU	4	Bekerja	1	Menikah	1	<Rp 2.500 a	1
SMU	4	Bekerja	1	Menikah	1	>RP 2.500 b	2
SD	2	Tidak Bek	2	Menikah	1	<Rp 2.500 a	1
SMU	4	Tidak Bek	2	Menikah	1	<Rp 2.500 a	1
SD	2	Tidak Bek	2	Menikah	1	<Rp 2.500 a	1
SLTP	3	Tidak Bek	2	Tidak Mer	2	<Rp 2.500 a	1

SMU	4	Tidak Bek	2	Menikah	1	<Rp 2.500 a	1
SLTP	3	Tidak Bek	2	Menikah	1	<Rp 2.500 a	1
SD	2	Bekerja	1	Menikah	1	<Rp 2.500 a	1
S1-S3	5	Bekerja	1	Menikah	1	>RP 2.500 b	2
S1-S3	5	Tidak Bek	2	Menikah	1	>RP 2.500 b	2
SMU	4	Tidak Bek	2	Menikah	1	<Rp 2.500 a	1
S1-S3	5	Tidak Bek	2	Menikah	1	<Rp 2.500 a	1
Tidak Sek	1	Bekerja	1	Menikah	1	<Rp 2.500 a	1
SMU	4	Bekerja	1	Menikah	1	>RP 2.500 b	2
SMU	4	Bekerja	1	Menikah	1	<Rp 2.500 a	1
S1-S3	5	Bekerja	1	Tidak Men	2	>RP 2.500 b	2
S1-S3	5	Bekerja	1	Menikah	1	>RP 2.500 b	2
SMU	4	Tidak Bek	2	Tidak Men	2	<Rp 2.500 a	1
SLTP	3	Tidak Bek	2	Menikah	1	<Rp 2.500 a	1
D3	5	Tidak Bek	2	Menikah	1	>RP 2.500 b	2
SMU	4	Tidak Bek	2	Menikah	1	>RP 2.500 b	2
SMU	4	Bekerja	1	Tidak Men	2	>RP 2.500 b	2
Tidak Sek	1	Tidak Bek	2	Tidak Men	2	<Rp 2.500 a	1
S1-S3	5	Bekerja	1	Menikah	1	>RP 2.500 b	2
SMU	4	Tidak Bek	2	Menikah	1	<Rp 2.500 a	1
SMU	4	Tidak Bek	2	Tidak Men	2	<RP 2.500 a	1
D3	5	Bekerja	1	Menikah	1	<Rp 2.500 a	1
SMU	4	Tidak Bek	2	Menikah	1	<RP 2.500 a	1
SMU	4	Bekerja	1	Tidak Men	2	<Rp 2.500 a	1
S1-S3	5	Bekerja	1	Tidak Men	2	<Rp 2.500 a	1
SD	2	Bekerja	1	Menikah	1	<Rp 2.500 a	1
SD	2	Bekerja	1	Menikah	1	<Rp 2.500 a	1
SMU	4	Bekerja	1	Menikah	1	>Rp 2.500 b	2
SMU	4	Bekerja	1	Menikah	1	<Rp 2.500 a	1
SD	2	Bekerja	1	Menikah	1	<Rp 2.500 a	1
D3	5	Tidak Bek	2	Menikah	1	>Rp 2.500 b	2
SD	2	Bekerja	1	Menikah	1	<Rp 2.500 a	1
SMU	4	Tidak Bek	2	Menikah	1	<Rp 2.500 a	1
SMU	4	Tidak Bek	2	Menikah	1	<Rp 2.500 a	1
SLTP	3	Bekerja	1	Menikah	1	>Rp 2.500 b	2
S1-S3	5	Bekerja	1	Menikah	1	>Rp 2.500 b	2
SLTP	3	Tidak Bek	2	Menikah	1	<Rp 2.500 a	1
SD	2	Tidak Bek	2	Menikah	1	<Rp 2.500 a	1
S1-S3	5	Bekerja	1	Menikah	1	<Rp 2.500 a	1
SLTP	3	Tidak Bek	2	Menikah	1	<Rp 2.500 a	1
SLTP	3	Tidak Bek	2	Menikah	1	<RP 2.500 a	1
SMU	4	Tidak Bek	2	Menikah	1	<RP 2.500 a	1
SD	2	Bekerja	1	Tidak Men	2	<RP 2.500 a	1
SD	2	Bekerja	1	Menikah	1	<RP 2.500 a	1
S1-S3	5	Tidak Bek	2	Menikah	1	>RP 2.500 b	2
D3	5	Bekerja	1	Menikah	1	>RP 2.500 b	2
SLTP	3	Tidak Bek	2	Menikah	1	<RP 2.500 a	1

SD	2	Bekerja	1	Tidak Mer	2	<RP 2.500 a	1
SD	2	Tidak Bek	2	Menikah	1	<RP 2.500 a	1
SD	2	Tidak Bek	2	Tidak Mer	2	>RP 2.500 b	2
SD	2	Bekerja	1	Menikah	1	<RP 2.500 a	1
SMU	4	Bekerja	1	Tidak Mer	2	<RP 2.500 a	1
D3	5	Tidak Bek	2	Menikah	1	<RP 2.500 a	1
SD	2	Bekerja	1	Menikah	1	<RP 2.500 a	1
SMU	4	Bekerja	1	Menikah	1	>RP 2.500 b	2
S1-S3	5	Tidak Bek	2	Menikah	1	>RP 2.500 b	2
SMU	4	Bekerja	1	Menikah	1	>RP 2.500 b	2
SMU	4	Bekerja	1	Menikah	1	<RP 2.500 a	1
S1-S3	5	Bekerja	1	Menikah	1	>RP 2.500 b	2
SMU	4	Bekerja	1	Menikah	1	<RP 2.500 a	1
SD	2	Tidak Bek	2	Menikah	1	>RP 2.500 b	2
SD	2	Tidak Bek	2	Menikah	1	<RP 2.500 a	1
SD	2	Bekerja	1	Menikah	1	<RP 2.500 a	1
SLTP	3	Tidak Bek	2	Menikah	1	<RP 2.500 a	1
SD	2	Tidak Bek	2	Menikah	1	<RP 2.500 a	1
SMU	4	Bekerja	1	Menikah	1	<RP 2.500 a	1
SMU	4	Bekerja	1	Tidak Mer	2	<RP 2.500 a	1
SMU	4	Tidak Bek	2	Menikah	1	>RP 2.500 b	2
SMU	4	Bekerja	1	Menikah	1	>RP 2.500 b	2
S1-S3	5	Tidak Bek	2	Menikah	1	<RP 2.500 a	1
D3	5	Tidak Bek	2	Menikah	1	<RP 2.500 a	1
SMU	4	Bekerja	1	Menikah	1	<RP 2.500 a	1
SD	2	Bekerja	1	Menikah	1	<RP 2.500 a	1
S1-S3	5	Tidak Bek	2	Menikah	1	>RP 2.500 b	2
SMU	4	Bekerja	1	Menikah	1	>RP 2.500 b	2
SD	2	Bekerja	1	Menikah	1	<RP 2.500 a	1
SD	2	Tidak Bek	2	Tidak Mer	2	<RP 2.500 a	1
SMU	4	Tidak Bek	2	Menikah	1	<RP 2.500 a	1
SMU	4	Tidak Bek	2	Menikah	1	<RP 2.500 a	1
SMU	4	Bekerja	1	Menikah	1	>RP 2.500 b	2
SMU	4	Tidak Bek	2	Tidak Mer	2	>RP 2.500 b	2
SD	2	Tidak Bek	2	Menikah	1	<RP 2.500 a	1
SD	2	Tidak Bek	2	Menikah	1	<RP 2.500 a	1
SD	2	Tidak Bek	2	Menikah	1	<RP 2.500 a	1
SMU	4	Tidak Bek	2	Menikah	1	<RP 2.500 a	1
SLTP	3	Bekerja	1	Menikah	1	<RP 2.500 a	1
S1-S3	5	Bekerja	1	Menikah	1	>RP 2.500 b	2
S1-S3	5	Bekerja	1	Menikah	1	<RP 2.500 a	1
SD	2	Tidak Bek	2	Menikah	1	<RP 2.500 a	1
S1-S3	5	Bekerja	1	Menikah	1	>RP 2.500 b	2
SMU	4	Tidak Bek	2	Tidak Mer	2	<RP 2.500 a	1
SMU	4	Tidak Bek	2	Menikah	1	<RP 2.500 a	1
SD	2	Bekerja	1	Menikah	1	<RP 2.500 a	1
S1-S3	5	Bekerja	1	Menikah	1	>RP 2.500 b	2

SMU	4	Bekerja	1	Menikah	1	<RP 2.500 a	1
SMU	4	Bekerja	1	Menikah	1	>RP 2.500 b	2
SLTP	3	Bekerja	1	Tidak Men	2	<Rp 2.500 a	1
SMU	4	Tidak Bek	2	Menikah	1	>RP 2.500 b	2
SLTP	3	Tidak Bek	2	Menikah	1	<Rp 2.500 a	1
SD	2	Tidak Bek	2	Menikah	1	<Rp 2.500 a	1
Tidak Sek	1	Tidak Bek	2	Menikah	1	>RP 2.500 b	2
SD	2	Tidak Bek	2	Menikah	1	<Rp 2.500 a	1
S1-S3	5	Bekerja	1	Menikah	1	>RP 2.500 b	2
SD	2	Tidak Bek	2	Menikah	1	<Rp 2.500 a	1
SMU	4	Tidak Bek	2	Menikah	1	<Rp 2.500 a	1
SMU	4	Bekerja	1	Menikah	1	>RP 2.500 b	2
SMU	4	Bekerja	1	Menikah	1	>RP 2.500 b	2
SMU	4	Tidak Bek	2	Menikah	1	<Rp 2.500 a	1
SD	2	Tidak Bek	2	Tidak Men	2	<Rp 2.500 a	1
S1-S3	5	Tidak Bek	2	Tidak Men	2	<Rp 2.500 a	1
SD	2	Tidak Bek	2	Tidak Men	2	<Rp 2.500 a	1
S1-S3	5	Bekerja	1	Menikah	1	>RP 2.500 b	2
SLTP	3	Tidak Bek	2	Menikah	1	<Rp 2.500 a	1
SMU	4	Tidak Bek	2	Menikah	1	>RP 2.500 b	2
SMU	4	Tidak Bek	2	Menikah	1	<Rp 2.500 a	1
SMU	4	Tidak Bek	2	Menikah	1	>RP 2.500 b	2
SLTP	3	Tidak Bek	2	Menikah	1	<Rp 2.500 a	1
S1-S3	5	Bekerja	1	Menikah	1	>RP 2.500 b	2
S1-S3	5	Bekerja	1	Menikah	1	>RP 2.500 b	2
SMU	4	Bekerja	1	Menikah	1	>RP 2.500 b	2
S1-S3	5	Tidak Bek	2	Menikah	1	>Rp 2.500 b	2
SD	2	Bekerja	1	Menikah	1	<RP 2.500 a	1
SD	2	Bekerja	1	Menikah	1	<Rp 2.500 a	1
SD	2	Tidak Bek	2	Menikah	1	<RP 2.500 a	1
SLTP	3	Tidak Bek	2	Menikah	1	<Rp 2.500 a	1
SMU	4	Tidak Bek	2	Menikah	1	<Rp 2.500 a	1
SLTP	3	Tidak Bek	2	Menikah	1	>Rp 2.500 b	2
SLTP	3	Tidak Bek	2	Menikah	1	<Rp 2.500 a	1
SLTP	3	Bekerja	1	Menikah	1	<Rp 2.500 a	1
Tidak Sek	1	Bekerja	1	Menikah	1	<Rp 2.500 a	1
SMU	4	Tidak Bek	2	Tidak Men	2	<Rp 2.500 a	1
SLTP	3	Tidak Bek	2	Tidak Men	2	<Rp 2.500 a	1
S1-S3	5	Bekerja	1	Menikah	1	>Rp 2.500 b	2
SLTP	3	Tidak Bek	2	Menikah	1	<Rp 2.500 a	1
SLTP	3	Tidak Bek	2	Tidak Men	2	<Rp 2.500 a	1
SD	2	Bekerja	1	Menikah	1	<Rp 2.500 a	1
SD	2	Bekerja	1	Menikah	1	<Rp 2.500 a	1
SMU	4	Tidak Bek	2	Tidak Men	2	>RP 2.500 b	2
SD	2	Bekerja	1	Menikah	1	<Rp 2.500 a	1
S1-S3	5	Bekerja	1	Menikah	1	>RP 2.500 b	2
SMU	4	Tidak Bek	2	Tidak Men	2	<Rp 2.500 a	1

SD	2	Bekerja	1	Menikah	1	<RP 2.500 a	1
SD	2	Tidak Bek	2	Menikah	1	<RP 2.500 a	1
SMU	4	Bekerja	1	Menikah	1	<RP 2.500 a	1
SD	2	Bekerja	1	Menikah	1	<RP 2.500 a	1
S1-S3	5	Tidak Bek	2	Menikah	1	<RP 2.500 a	1
SD	2	Bekerja	1	Tidak Men	2	<RP 2.500 a	1
SD	2	Bekerja	1	Menikah	1	>RP 2.500 b	2
SMU	4	Tidak Bek	2	Menikah	1	<RP 2.500 a	1
SD	2	Tidak Bek	2	Tidak Men	2	<RP 2.500 a	1
Tidak Sek	1	Tidak Bek	2	Menikah	1	>RP 2.500 b	2
SD	2	Bekerja	1	Menikah	1	<RP 2.500 a	1
S1-S3	5	Bekerja	1	Tidak Men	2	>RP 2.500 b	2
SD	2	Tidak Bek	2	Tidak Men	2	<RP 2.500 a	1
SLTP	3	Tidak Bek	2	Menikah	1	<RP 2.500 a	1
SD	2	Tidak Bek	2	Menikah	1	<RP 2.500 a	1
S1-S3	5	Tidak Bek	2	Menikah	1	>RP 2.500 b	2
SD	2	Tidak Bek	2	Menikah	1	<RP 2.500 a	1
SLTP	3	Bekerja	1	Tidak Men	2	<RP 2.500 a	1
Tidak Sek	1	Bekerja	1	Tidak Men	2	<RP 2.500 a	1
S1-S3	5	Bekerja	1	Menikah	1	>RP 2.500 b	2
S1-S3	5	Tidak Bek	2	Menikah	1	<RP 2.500 a	1
SD	2	Tidak Bek	2	Menikah	1	<RP 2.500 a	1
S1-S3	5	Tidak Bek	2	Menikah	1	<RP 2.500 a	1
D3	5	Tidak Bek	2	Menikah	1	>RP 2.500 b	2
S1-S3	5	Bekerja	1	Menikah	1	<RP 2.500 a	1
SD	2	Bekerja	1	Menikah	1	<RP 2.500 a	1
SLTP	3	Tidak Bek	2	Menikah	1	>RP 2.500 b	2
Tidak Sek	1	Bekerja	1	Menikah	1	<RP 2.500 a	1
SMU	4	Tidak Bek	2	Menikah	1	>RP 2.500 b	2
SMU	4	Bekerja	1	Menikah	1	<RP 2.500 a	1
SMU	4	Bekerja	1	Menikah	1	<RP 2.500 a	1
SMU	4	Bekerja	1	Menikah	1	<RP 2.500 a	1
S1-S3	5	Tidak Bek	2	Menikah	1	>RP 2.500 b	2
SD	2	Bekerja	1	Tidak Men	2	<RP 2.500 a	1
SD	2	Tidak Bek	2	Tidak Men	2	<RP 2.500 a	1
SMU	4	Tidak Bek	2	Tidak Men	2	<RP 2.500 a	1
SD	2	Bekerja	1	Menikah	1	<RP 2.500 a	1
SMU	4	Tidak Bek	2	Menikah	1	<RP 2.500 a	1
SMU	4	Tidak Bek	2	Menikah	1	>RP 2.500 b	2
SD	2	Tidak Bek	2	Menikah	1	<RP 2.500 a	1
SD	2	Tidak Bek	2	Menikah	1	<RP 2.500 a	1
S1-S3	5	Bekerja	1	Menikah	1	>RP 2.500 b	2
SD	2	Bekerja	1	Menikah	1	<RP 2.500 a	1
SMU	4	Tidak Bek	2	Tidak Men	2	<RP 2.500 a	1
Tidak Sek	1	Tidak Bek	2	Menikah	1	<RP 2.500 a	1
SLTP	3	Bekerja	1	Menikah	1	<RP 2.500 a	1
SMU	4	Bekerja	1	Menikah	1	<RP 2.500 a	1

SMU	4	Bekerja	1	Menikah	1	>RP 2.500 b	2
S1-S3	5	Tidak Bek	2	Menikah	1	>RP 2.500 b	2
SD	2	Bekerja	1	Menikah	1	<RP 2.500 a	1
SMU	4	Tidak Bek	2	Tidak Mer	2	>RP 2.500 b	2
Tidak Sek	1	Bekerja	1	Menikah	1	<RP 2.500 a	1
SD	2	Bekerja	1	Menikah	1	<RP 2.500 a	1
SMU	4	Bekerja	1	Menikah	1	<RP 2.500 a	1
SMU	4	Tidak Bek	2	Tidak Mer	2	<RP 2.500 a	1
SLTP	3	Bekerja	1	Menikah	1	<Rp 2.500 a	1
SMU	4	Tidak Bek	2	Menikah	1	<Rp 2.500 a	1
SLTP	3	Tidak Bek	2	Menikah	1	<Rp 2.500 a	1
SD	2	Tidak Bek	2	Menikah	1	<Rp 2.500 a	1
S1-S3	5	Tidak Bek	2	Tidak Mer	2	>RP 2.500 b	2
SLTP	3	Bekerja	1	Tidak Mer	2	<Rp 2.500 a	1
S1-S3	5	Bekerja	1	Menikah	1	>RP 2.500 b	2
SMU	4	Tidak Bek	2	Tidak Mer	2	>RP 2.500 b	2
SMU	4	Tidak Bek	2	Menikah	1	<Rp 2.500 a	1
S1-S3	5	Bekerja	1	Menikah	1	>RP 2.500 b	2
SMU	4	Bekerja	1	Menikah	1	<RP 2.500 a	1
SD	2	Tidak Bek	2	Tidak Mer	2	<RP 2.500 a	1
SMU	4	Tidak Bek	2	Menikah	1	>RP 2.500 b	2
SD	2	Tidak Bek	2	Menikah	1	>RP 2.500 b	2
S1-S3	5	Tidak Bek	2	Menikah	1	>RP 2.500 b	2
S1-S3	5	Bekerja	1	Menikah	1	>RP 2.500 b	2
SD	2	Tidak Bek	2	Menikah	1	<RP 2.500 a	1
D3	5	Tidak Bek	2	Menikah	1	>RP 2.500 b	2
SD	2	Tidak Bek	2	Menikah	1	<RP 2.500 a	1
SD	2	Bekerja	1	Menikah	1	>RP 2.500 b	2
SLTP	3	Tidak Bek	2	Menikah	1	>RP 2.500 b	2
SMU	4	Bekerja	1	Menikah	1	>RP 2.500 b	2
SD	2	Tidak Bek	2	Menikah	1	>RP 2.500 b	2
SMU	4	Tidak Bek	2	Menikah	1	>Rp 2.500 b	2
S1-S3	5	Tidak Bek	2	Menikah	1	>Rp 2.500 b	2
SMU	4	Bekerja	1	Tidak Mer	2	>Rp 2.500 b	2
Tidak Sek	1	Tidak Bek	2	Tidak Mer	2	<RP 2.500 a	1
SD	2	Bekerja	1	Menikah	1	<RP 2.500 a	1
SMU	4	Bekerja	1	Menikah	1	>RP 2.500 b	2
SMU	4	Tidak Bek	2	Menikah	1	>RP 2.500 b	2
S1-S3	5	Bekerja	1	Menikah	1	>RP 2.500 b	2
SMU	4	Bekerja	1	Tidak Mer	2	<RP 2.500 a	1
SD	2	Bekerja	1	Menikah	1	<RP 2.500 a	1
S1-S3	5	Bekerja	1	Menikah	1	>RP 2.500 b	2
SMU	4	Bekerja	1	Menikah	1	<RP 2.500 a	1
SMU	4	Bekerja	1	Menikah	1	<RP 2.500 a	1
SLTP	3	Tidak Bek	2	Menikah	1	<RP 2.500 a	1
SMU	4	Tidak Bek	2	Menikah	1	>RP 2.500 b	2
S1-S3	5	Tidak Bek	2	Menikah	1	>RP 2.500 b	2

SD	2	Bekerja	1	Menikah	1	<RP 2.500	a	1
SLTP	3	Bekerja	1	Menikah	1	<RP 2.500	a	1
S1-S3	5	Bekerja	1	Menikah	1	>RP 2.500	b	2
Tidak Sek	1	Tidak Bek	2	Tidak Mer	2	<RP 2.500	a	1
SMU	4	Tidak Bek	2	Menikah	1	<RP 2.500	a	1
S1-S3	5	Tidak Bek	2	Menikah	1	>RP 2.500	b	2
SMU	4	Bekerja	1	Menikah	1	>RP 2.500	b	2
D3	5	Tidak Bek	2	Menikah	1	>RP 2.500	b	2
S1-S3	5	Bekerja	1	Menikah	1	>RP 2.500	b	2
SMU	4	Tidak Bek	2	Menikah	1	<RP 2.500	a	1
SMU	4	Bekerja	1	Menikah	1	<RP 2.500	a	1
SLTP	3	Tidak Bek	2	Menikah	1	<RP 2.500	a	1
SD	2	Bekerja	1	Menikah	1	<RP 2.500	a	1
S1-S3	5	Bekerja	1	Menikah	1	>RP 2.500	b	2
SLTP	3	Bekerja	1	Menikah	1	<RP 2.500	a	1
S1-S3	5	Bekerja	1	Menikah	1	>RP 2.500	b	2
D3	5	Tidak Bek	2	Menikah	1	>RP 2.500	b	2
S1-S3	5	Tidak Bek	2	Menikah	1	>RP 2.500	b	2
D3	5	Tidak Bek	2	Tidak Mer	2	>RP 2.500	b	2
SMU	4	Tidak Bek	2	Menikah	1	<Rp 2.500	b	2
S1	5	Bekerja	1	Menikah	1	>RP 2.50	a	1
SD	2	Tidak Bek	2	Menikah	1	<Rp 2.500	b	2
SMU	4	Tidak Bek	2	Menikah	1	<Rp 2.500	b	2
SMP	3	Bekerja	1	Menikah	1	<Rp 2.500	b	2
SMU	4	Tidak Bek	2	Menikah	1	<Rp 2.500	b	2
SD	2	Tidak Bek	2	Menikah	1	<Rp 2.500	b	2
SD	2	Tidak Bek	2	Menikah	1	<Rp 2.500	b	2
Tidak Sek	1	Bekerja	1	Menikah	1	<Rp 2.500	b	2
SD	2	Tidak Bek	2	Menikah	1	<Rp 2.500	b	2
S1	5	Bekerja	1	Menikah	1	>RP 2.50	a	1
SD	2	Tidak Bek	2	Menikah	1	<Rp 2.500	b	2
SMU	4	Tidak Bek	2	Menikah	1	<Rp 2.500	b	2
SMU	4	Tidak Bek	2	Tidak Mer	2	<Rp 2.500	b	2
SD	2	Tidak Bek	2	Menikah	1	<Rp 2.500	b	2
SMU	4	Tidak Bek	2	Menikah	1	<Rp 2.500	b	2
SMU	4	Tidak Bek	2	Tidak Mer	2	<Rp 2.500	b	2
SLTP	3	Tidak Bek	2	Menikah	1	<Rp 2.500	b	2
SD	2	Tidak Bek	2	Menikah	1	<Rp 2.500	b	2
S1	5	Bekerja	1	Menikah	1	>Rp 2.500	b	2
SMU	4	Tidak Bek	2	Menikah	1	<Rp 2.500	b	2
SD	2	Tidak Bek	2	Menikah	1	<Rp 2.500	b	2
SMU	4	Bekerja	1	Menikah	1	<Rp 2.500	b	2
SLTP	3	Tidak Bek	2	Menikah	1	<Rp 2.500	b	2
S1	5	Tidak Bek	2	Menikah	1	<Rp 2.500	b	2
SD	2	Tidak Bek	2	Menikah	1	<Rp 2.500	b	2
SD	2	Bekerja	1	Menikah	1	<RP 2.50	b	2
SD	2	Tidak Bek	2	Menikah	1	<RP 2.50	b	2

SMU	4	Tidak Bek	2	Menikah	1	<RP 2.50 b	2
D3	5	Tidak Bek	2	Menikah	1	<RP 2.50 b	2
SD	2	Tidak Bek	2	Menikah	1	<RP 2.50 b	2
SMU	4	Tidak Bek	2	Menikah	1	<RP 2.50 b	2
SLTP	3	Tidak Bek	2	Menikah	1	<RP 2.50 b	2
SLTP	3	Tidak Bek	2	Menikah	1	<RP 2.50 b	2
SLTP	3	Tidak Bek	2	Menikah	1	<RP 2.50 b	2
S1	5	Bekerja	1	Menikah	1	>RP 2.50 a	1
SD	2	Tidak Bek	2	Menikah	1	<RP 2.50 b	2
SD	2	Tidak Bek	2	Menikah	1	<RP 2.50 b	2
Tidak Sek	1	Tidak Bek	2	Menikah	1	<RP 2.50 b	2
SD	2	Tidak Bek	2	Menikah	1	<RP 2.50 b	2
SMU	4	Tidak Bek	2	Menikah	1	<RP 2.50 b	2
SD	2	Tidak Bek	2	Menikah	1	<RP 2.50 b	2
SD	2	Tidak Bek	2	Menikah	1	<RP 2.50 b	2
SD	2	Tidak Bek	2	Menikah	1	<RP 2.50 b	2
SLTP	3	Tidak Bek	2	Menikah	1	<RP 2.50 b	2
SMU	4	Bekerja	1	Menikah	1	<RP 2.50 b	2
SD	2	Tidak Bek	2	Menikah	1	<RP 2.50 b	2
SD	2	Tidak Bek	2	Menikah	1	<RP 2.50 b	2
SD	2	Tidak Bek	2	Menikah	1	<RP 2.50 b	2
SD	2	Bekerja	1	Menikah	1	<RP 2.50 b	2
S1	5	Tidak Bek	2	Menikah	1	<RP 2.50 b	2
SD	2	Tidak Bek	2	Menikah	1	<RP 2.50 b	2
SD	2	Bekerja	1	Menikah	1	<RP 2.50 b	2
SD	2	Tidak Bek	2	Menikah	1	<RP 2.50 b	2
SD	2	Tidak Bek	2	Menikah	1	<RP 2.50 b	2
SLTP	3	Bekerja	1	Menikah	1	<RP 2.50 b	2
SD	2	Tidak Bek	2	Menikah	1	<RP 2.50 b	2
SMU	4	Tidak Bek	2	Menikah	1	<RP 2.50 b	2
SD	2	Tidak Bek	2	Menikah	1	<Rp 2.50(b	2
SD	2	Tidak Bek	2	Menikah	1	<RP 2.50 b	2
SD	2	Tidak Bek	2	Menikah	1	<RP 2.50 b	2
SLTP	3	Bekerja	1	Menikah	1	<RP 2.50 b	2
SMU	4	Bekerja	1	Menikah	1	<RP 2.50 b	2
SD	2	Bekerja	1	Menikah	1	<RP 2.50 b	2
SMU	4	Tidak Bek	2	Menikah	1	<RP 2.50 b	2
Tidak Sek	1	Tidak Bek	2	Menikah	1	<RP 2.50 b	2
SMU	4	Tidak Bek	2	Menikah	1	<RP 2.50 b	2
SD	2	Tidak Bek	2	Menikah	1	<RP 2.50 b	2
SD	2	Tidak Bek	2	Menikah	1	<RP 2.50 b	2
S1	5	Bekerja	1	Menikah	1	>RP 2.50 a	1
SMU	4	Tidak Bek	2	Menikah	1	<RP 2.50 b	2
SMU	4	Tidak Bek	2	Menikah	1	<RP 2.50 b	2
SD	2	Tidak Bek	2	Menikah	1	<RP 2.50 b	2
SLTP	3	Tidak Bek	2	Menikah	1	<RP 2.50 b	2
SD	2	Tidak Bek	2	Menikah	1	<RP 2.50 b	2

SLTP	3	Tidak Bek	2	Menikah	1	<RP 2.50 b	2
SD	2	Tidak Bek	2	Menikah	1	<RP 2.50 b	2
SD	2	Tidak Bek	2	Menikah	1	<Rp 2.50 b	2
SMU	4	Tidak Bek	2	Menikah	1	<Rp 2.50 b	2
SMU	4	Tidak Bek	2	Menikah	1	<Rp 2.50 b	2
SMU	4	Tidak Bek	2	Menikah	1	<Rp 2.50 b	2
SLTP	3	Tidak Bek	2	Menikah	1	<RP 2.50 b	2
SD	2	Tidak Bek	2	Menikah	1	<RP 2.50 b	2
SD	2	Tidak Bek	2	Menikah	1	<RP 2.50 b	2
SMU	4	Tidak Bek	2	Menikah	1	<RP 2.50 b	2
SLTP	3	Tidak Bek	2	Menikah	1	<RP 2.50 b	2
SLTP	3	Tidak Bek	2	Menikah	1	<RP 2.50 b	2
SD	2	Tidak Bek	2	Menikah	1	<RP 2.50 b	2
SD	2	Tidak Bek	2	Menikah	1	<RP 2.50 b	2
S1-S3	5	Tidak Bek	2	Menikah	1	<RP 2.50 b	2
SLTP	3	Tidak Bek	2	Menikah	1	<RP 2.50 b	2
SMU	4	Tidak Bek	2	Menikah	1	<RP 2.50 b	2
S1-S3	5	Tidak Bek	2	Menikah	1	<RP 2.50 b	2
SLTP	3	Bekerja	1	Menikah	1	<RP 2.50 b	2
SD	2	Tidak Bek	2	Menikah	1	<RP 2.50 b	2
SD	2	Bekerja	1	Menikah	1	<RP 2.50 b	2
SD	2	Tidak Bek	2	Menikah	1	<RP 2.50 b	2
SD	2	Tidak Bek	2	Menikah	1	<RP 2.50 b	2
Tidak Sek	1	Tidak Bek	2	Menikah	1	<RP 2.50 b	2
SMU	4	Tidak Bek	2	Menikah	1	<RP 2.50 b	2
SD	2	Tidak Bek	2	Menikah	1	<RP 2.50 b	2
Tidak Sek	1	Tidak Bek	2	Menikah	1	<RP 2.50 b	2
SMU	4	Tidak Bek	2	Menikah	1	<RP 2.50 b	2
Tidak Sek	1	Tidak Bek	2	Menikah	1	<RP 2.50 b	2
SD	2	Tidak Bek	2	Menikah	1	<RP 2.50 b	2
SD	2	Tidak Bek	2	Menikah	1	<RP 2.50 b	2
SMU	4	Tidak Bek	2	Menikah	1	<RP 2.50 b	2
SD	2	Tidak Bek	2	Menikah	1	<RP 2.50 b	2
SD	2	Tidak Bek	2	Menikah	1	<RP 2.50 b	2
SMU	4	Tidak Bek	2	Menikah	1	<RP 2.50 b	2
SMU	4	Bekerja	1	Menikah	1	<RP 2.50 b	2
SD	2	Tidak Bek	2	Menikah	1	<RP 2.50 b	2
SD	2	Tidak Bek	2	Menikah	1	<RP 2.50 b	2
SLTP	3	Tidak Bek	2	Menikah	1	<RP 2.50 b	2
SMU	4	Tidak Bek	2	Menikah	1	<RP 2.50 b	2
S1-S3	5	Bekerja	1	Menikah	1	>RP 2.50 a	1
SMU	4	Tidak Bek	2	Menikah	1	<RP 2.50 b	2
SD	2	Tidak Bek	2	Menikah	1	<RP 2.50 b	2
SLTP	3	Tidak Bek	2	Menikah	1	<RP 2.50 b	2
SD	2	Tidak Bek	2	Menikah	1	<RP 2.50 b	2
SMU	4	Tidak Bek	2	Menikah	1	<RP 2.50 b	2

SD	2	Tidak Bek	2	Menikah	1	<RP 2.50 b	2
SLTP	3	Tidak Bek	2	Menikah	1	<RP 2.50 b	2
SMU	4	Tidak Bek	2	Menikah	1	<RP 2.50 b	2
SD	2	Tidak Bek	2	Menikah	1	<RP 2.50 b	2
SD	2	Tidak Bek	2	Menikah	1	<RP 2.50 b	2
S1-S3	5	Bekerja	1	Menikah	1	<RP 2.50 b	2
SD	2	Tidak Bek	2	Menikah	1	<RP 2.50 b	2
SD	2	Tidak Bek	2	Menikah	1	<RP 2.50 b	2
SMU	4	Tidak Bek	2	Menikah	1	<RP 2.50 b	2
Tidak Sek	1	Tidak Bek	2	Menikah	1	<RP 2.50 b	2
SD	2	Tidak Bek	2	Menikah	1	<RP 2.50 b	2
SD	2	Tidak Bek	2	Menikah	1	<RP 2.50 b	2
Tidak Sek	1	Bekerja	1	Menikah	1	<RP 2.50 b	2
SMU	4	Tidak Bek	2	Menikah	1	<RP 2.50 b	2
D3	5	Tidak Bek	2	Menikah	1	<RP 2.50 b	2
SLTP	3	Tidak Bek	2	Menikah	1	<RP 2.50 b	2
SD	2	Bekerja	1	Menikah	1	<RP 2.50 b	2
SMU	4	Tidak Bek	2	Menikah	1	<RP 2.50 b	2
SD	2	Tidak Bek	2	Menikah	1	<RP 2.50 b	2
SD	2	Tidak Bek	2	Menikah	1	<RP 2.50 b	2
SMU	4	Tidak Bek	2	Menikah	1	<RP 2.50 b	2
SD	2	Tidak Bek	2	Menikah	1	<RP 2.50 b	2
S1-S3	5	Bekerja	1	Menikah	1	<RP 2.50 b	2
SD	2	Tidak Bek	2	Menikah	1	<RP 2.50 b	2
D3	5	Tidak Bek	2	Menikah	1	<RP 2.50 b	2
SD	2	Tidak Bek	2	Menikah	1	<RP 2.50 b	2
SMU	4	Tidak Bek	2	Menikah	1	<RP 2.50 b	2
SD	2	Tidak Bek	2	Menikah	1	<RP 2.50 b	2
SD	2	Tidak Bek	2	Menikah	1	<RP 2.50 b	2
SD	2	Tidak Bek	2	Menikah	1	<RP 2.50 b	2
